# Associations of Cumulative Adulthood, Childhood and Lifelong Insulin With Adulthood Retinal Microvasculature

**DOI:** 10.1210/clinem/dgae865

**Published:** 2024-12-11

**Authors:** Oskari Repo, Markus Juonala, Suvi P Rovio, Juha Mykkänen, Jaakko Nevalainen, Mika Kähönen, Terho Lehtimäki, Tomi P Laitinen, Jorma Viikari, Olli Raitakari, Robyn Tapp, Katja Pahkala

**Affiliations:** Research Centre of Applied and Preventive Cardiovascular Medicine, University of Turku, 20520 Turku, Finland; Centre for Population Health Research, University of Turku and Turku University Hospital, 20520 Turku, Finland; Department of Medicine, University of Turku, 20520 Turku, Finland; Division of Medicine, Turku University Hospital, 20520 Turku, Finland; Research Centre of Applied and Preventive Cardiovascular Medicine, University of Turku, 20520 Turku, Finland; Centre for Population Health Research, University of Turku and Turku University Hospital, 20520 Turku, Finland; Department of Public Health, University of Turku and Turku University Hospital, 20520 Turku, Finland; Research Centre of Applied and Preventive Cardiovascular Medicine, University of Turku, 20520 Turku, Finland; Centre for Population Health Research, University of Turku and Turku University Hospital, 20520 Turku, Finland; Unit of Health Sciences, Faculty of Social Sciences, Tampere University, 33520 Tampere, Finland; Department of Clinical Physiology, Tampere University Hospital and Faculty of Medicine and Health Technology, Tampere University, 33521 Tampere, Finland; Department of Clinical Chemistry, Fimlab Laboratories, and Finnish Cardiovascular Research Center-Tampere, Faculty of Medicine and Health Technology, Tampere University, 33521 Tampere, Finland; Department of Clinical Physiology, University of Eastern Finland and Kuopio University Hospital, 70029 Kuopio, Finland; Department of Medicine, University of Turku, 20520 Turku, Finland; Division of Medicine, Turku University Hospital, 20520 Turku, Finland; Research Centre of Applied and Preventive Cardiovascular Medicine, University of Turku, 20520 Turku, Finland; Centre for Population Health Research, University of Turku and Turku University Hospital, 20520 Turku, Finland; Department of Clinical Physiology and Nuclear Medicine, Turku University Hospital, 20014 Turku, Finland; InFLAMES Research Flagship, University of Turku, 20014 Turku, Finland; Melbourne School of Population and Global Health, University of Melbourne, Melbourne, VIC 3010, Australia; Centre for Intelligent Health Care, Coventry University, Coventry CV1 2TU, UK; Research Centre of Applied and Preventive Cardiovascular Medicine, University of Turku, 20520 Turku, Finland; Centre for Population Health Research, University of Turku and Turku University Hospital, 20520 Turku, Finland; Paavo Nurmi Centre & Unit for Health and Physical Activity, University of Turku, 20520 Turku, Finland

**Keywords:** insulin, microvascular changes, arterioles, retina, lifespan

## Abstract

**Context:**

Exogenous insulin is reported to have both vasodilatory and vasoconstrictive effects on microvasculature. Little is known about the associations of long-term endogenous insulin exposure with microvasculature.

**Objective:**

To test the hypothesis that long-term exposure to high insulin levels in childhood and adulthood is associated with adverse changes in retinal microvasculature in adulthood in a population without diabetes.

**Methods:**

We analyzed data derived from the longitudinal Cardiovascular Risk in Young Finns Study. The first cross-sectional study was conducted in 1980, and participants were followed for 31 years from childhood to adulthood with frequent follow-up visits. Fundus photos were taken in 2011, and microvascular outcome measures were derived in participants at the age of 34 to 49 years (n = 1684). After exclusion of individuals with diabetes or missing insulin measures, 1166 participants formed the population of the present study. Cumulative exposure as the area under the curve (AUC) for adulthood (10-year exposure between 2001 and 2011) and childhood (exposure between ages 6-18 years) insulin and other cardiovascular risk factors were determined. Additionally, adulthood and childhood cumulative AUCs were summarized to construct lifelong AUCs.

**Results:**

Higher adulthood, childhood, and lifelong exposure for cumulative insulin was associated with decreased retinal arteriolar diameter when adjusted for age and sex and further for cumulative conventional cardiovascular risk factors.

**Conclusion:**

Cumulative childhood, adulthood, and lifelong insulin are associated with decreased retinal arteriolar diameter in adulthood in a population of participants without diabetes, independently of conventional cardiovascular risk factors.

A Western lifestyle, characterized by an energy-rich diet and sedentary lifestyle, is leading to an increasing prevalence of metabolic disorders globally (1). The pathophysiology of metabolic disorders seems to be largely attributable to insulin resistance (2). Insulin resistance and accompanying hyperinsulinemia play a pivotal role in the transition from a metabolic disease to cardiovascular disease development (3), and reciprocal relationships between insulin resistance and endothelial dysfunction provide a pathophysiological mechanism connecting disorders of metabolic and cardiovascular homeostasis (4). It has been previously hypothesized that endothelial dysfunction has downstream effects on microvascular structure and function (5), and endothelial dysfunction of the microvasculature may be one of the first steps of the cascade leading to cardiovascular disease progression (5, 6). Therefore, studying microvasculature offers an intriguing approach to early vascular changes related to insulin resistance.

Previously, studies on the effects of exogenous insulin, mostly on the microvasculature of skeletal muscle, have reported that in a metabolically healthy population, insulin has a vasodilatory effect on microvasculature (7-9). In contrast, in participants defined as having metabolic insulin resistance (obesity or metabolic syndrome), the effect of insulin seems to be vasoconstrictive, leading to impaired blood flow and glucose uptake. Based on these findings, it has been hypothesized that in the state of insulin resistance, the beneficial metabolic effects of insulin are converted to adverse effects (4, 9).

Retinal microvasculature shares numerous characteristics with systemic and coronary microcirculation. Therefore, retinal photography offers a noninvasive approach to microvasculature study and is described as a window to the heart (10). Indeed, fundus photo-derived retinal microvascular parameters, such as diameters and tortuosity, are predictive of cardiovascular morbidity and mortality (11-13). These retinal parameters are considered to reflect the retinal microvascular integrity, which incorporates both structural and functional components (6).

In addition to previous studies on microvascular effects of exogenous insulin (9), studies on associations between long-term endogenous insulin and retinal microvasculature could improve our understanding of microvascular integrity (functional and structural) changes associated with insulin and insulin resistance. We have recently reported in the STRIP cohort following individuals from infancy to age 26 years that cumulative insulin from childhood to early adulthood was associated with adverse microvascular integrity and structure characterized by narrower arterioles and more tortuous venules in early adulthood (14).

In this study, we leveraged data from the Cardiovascular Risk in Young Finns Study (YFS) to investigate the associations of cumulative insulin exposure with retinal microvasculature in a larger, middle-aged population and to utilize cumulative insulin data from both childhood and adulthood. Additionally, to test the hypothesis that cumulative endogenous insulin is adversely associated with microvascular integrity only in the presence of metabolic insulin resistance, as suggested by experimental studies investigating the microvascular effects of exogenous insulin (7, 8), we further study whether the associations of cumulative adulthood insulin with retinal microvasculature are different in those participants with clustering of metabolic risk factors (“metabolic syndrome”) and those defined as being metabolically healthy.

## Materials and Methods

YFS is an ongoing prospective multicenter study from Finland initiated in the late 1970s (15). The study has been carried out in all 5 Finnish university cities with medical schools and their rural surroundings. The first cross-sectional study was conducted in 1980. Altogether, 4320 children and adolescents aged 3, 6, 9, 12, 15, and 18 years were randomly chosen from the population register of these areas to produce a representative sample of Finnish children. In practice, females and males of each age cohort in each study community were separately placed in random order on the basis of the unique personal identification number. Every *k*th female and every *k*th male in each community was selected so that the sample consisted of the required number of males and females. The varying *k* factors were determined on the basis of sample size and the total number of males and females in the different age cohorts in each community. The cohort has been followed at regular intervals in 1983, 1986, 2001, 2007, and 2011. Detailed descriptions on the population and protocols are previously reported (15). Loss to follow-up at adult follow-ups has also been previously reported (16); briefly, when compared to nonparticipants, participants were more often females and older than nonparticipants with no significant differences in modifiable cardiovascular risk factors at the baseline.

**Figure 1.**
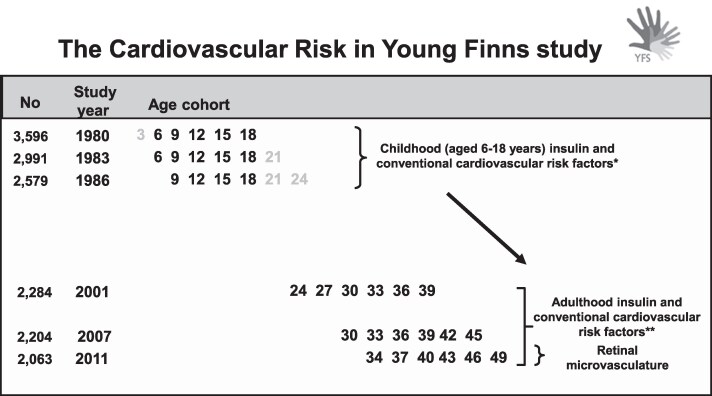
Design of the present study. The cumulative insulin and conventional cardiovascular risk factor exposures were calculated from childhood and adulthood as under the curve. In the 2011 follow-up, fundus imaging was performed, and adulthood microvascular outcome measures were derived. *Insulin measurements from 1980 to 1986 were utilized to assess the childhood (aged 6-18 years) cumulative insulin exposure. ** Insulin measurements from 2001 to 2011 were utilized to assess 10-year adulthood cumulative insulin exposure.

In 2011, fundus imaging was performed (n = 1684), and 1676 participants provided gradable retinal data for arteriolar and venular measures. Of these participants, 1212 provided data on fasting insulin from all 3 timepoints in adulthood (follow-up years 2001, 2007, and 2011) required to assess 10-year adulthood cumulative insulin load. Participants with type 1 diabetes were excluded from the study (n = 5). Regarding type 2 diabetes, cardiometabolic risk factor associations with retinal microvasculature have been reported to differ by the diabetes status (17). Because of this, and their limited number, participants with type 2 diabetes were also excluded (n = 41). Overall, 1166 participants met the inclusion criteria, and these participants formed the here-applied cohort. Data from these participants were further used to study associations of childhood cumulative insulin (for age 6-18 years, n = 1166) with the retinal microvasculature. The design of the present study is illustrated in Fig. 1. The study was approved by local ethics committees (Ethics Committee, Hospital District of Southwest Finland). All participants provided written informed consent.

### Retinal Measurements

Forty-five-degree digital retinal images were captured in 2011 using a Canon nonmydriatic retinal camera (Canon CR6-45NM, USA) fitted with a canon 10D digital SLR camera (resolution 3072 × 2048 pixels). Images were centered on the macula of each eye. In this study, 1 pixel corresponds to approximately 5 μm, and the estimated diameter of arterioles was ∼100 μm. A semiautomated grading system was used to capture a range of vascular geometric parameters including

- Arteriolar and venular diameters
- Arteriolar and venular tortuosity; estimated as the actual length of the vessel divided by the straight-line distance between bifurcations, minus 1

The arteriolar and venular diameters were measured at a series of cross-sections normal to the vessel at 2-pixel intervals along the entire length of the vessel segment. At each cross-section, the vessel diameter was measured to subpixel accuracy using a sliding linear regression filter technique as described previously and an average calculated for each vessel (18). Images were read at a single reading center (Imperial College London), and photographer accreditation was performed prior to beginning the study. Imaging quality control was conducted regularly throughout the study. One observer, blinded to subject data, undertook quality control, including provision of feedback, and performed retinal grading. The reproducibility of this technique has been reported previously (18-21). In the present study, the reproducibility of this technique was excellent (intraclass correlation coefficients for within-observer measurements were >0.9), and the average absolute difference and SD between measurements of arteriolar diameter was 0.0 ± 0.4 pixels, consistent with previous reports.

### Insulin and Other Conventional Cardiovascular Risk Factors

Standard methods were used for measuring insulin and other conventional risk factors: serum insulin, total cholesterol, high-density lipoprotein (HDL) cholesterol, triglycerides, and blood pressure were measured at baseline (1980) and at all follow-up studies. Details of these methods have been described previously (15, 22). In the years 1980, 1983, and 1986, serum insulin levels were measured with a modification of the immunoassay method of Herbert et al (23, 24). In the years 2001, 2007, and 2011, serum insulin levels were measured by a microparticle enzyme immunoassay kit (Abbott Laboratories, Diagnostic Division, Dainabot, Catalog # 8K41-74, RRID: AB_3075437) (24). All laboratory measurements were from fasting serum samples. Low-density lipoprotein (LDL) cholesterol was calculated according to Friedewald (25). Serum glucose was measured since the 1986 follow-up. At all follow-ups, weight and height were measured and body mass index (BMI) was calculated (26).

Data on smoking was collected via questionnaires. Adulthood smoking was defined as daily smoking (yes/no) at the 2011 follow-up. Childhood smoking was defined as daily smoking (yes/no) at baseline or at any follow-up visit when the participants were aged 12 to 18 years.

### Adulthood Cumulative Insulin and Other Conventional Cardiovascular Risk Factors

To assess cumulative exposure to insulin in adulthood, we used linear interpolation between 3 consecutive measurements and evaluated the area under the curve (AUC) to construct the cumulative exposure variable. Similarly, we constructed an AUC variable for cumulative systolic blood pressure, BMI, LDL-cholesterol, HDL-cholesterol, triglycerides, and glucose. The approach is similar to Huang et al (27), even though we used absolute compared to time-averaged values for the AUCs to evaluate cumulative rather than long-term average risk factor load. For the calculation of the AUCs, data on the risk factors were required to be available from all 3 adulthood follow-up visits (2001, 2007, 2011). For interpretability, the adulthood insulin AUC was standardized resulting in a variable with a mean 0 and SD 1; thus the β coefficients indicate a change in the retinal variables when the cumulative insulin exposure increases by 1 SD.

### Childhood Cumulative Insulin and Other Conventional Cardiovascular Risk Factors

In addition to assessing 10-year cumulative adulthood insulin exposure, we were able to assess cumulative exposure in childhood. However, due to the study design and differences in the available number of measures for each age group in childhood (see Fig. 1), a different method was utilized in constructing AUC variables in childhood. Subject-specific curves for insulin and other cardiovascular risk factors were estimated by mixed model regression splines (28), similarly to Rovio et al. (29), to use all available repeatedly collected exposure data from childhood. This method allowed us to construct a childhood risk factor AUC for every participant despite missing observations. Mixed model regression splines can handle incomplete sequences of observations and provide valid estimates under the missing at random mechanism. The childhood AUC variables were defined for age 6 to 18 years (9-18 for glucose), and they were standardized (mean 0, SD 1).

### Lifelong Cumulative Insulin and Other Conventional Cardiovascular Risk Factors

To assess combined lifelong cumulative insulin exposure, the standardized adulthood and childhood insulin AUCs were summed. To make the combined lifelong cumulative insulin AUC comparable with the adulthood and childhood cumulative insulin AUCs, the combined lifelong insulin AUC was also standardized (mean 0, SD 1); thus the β coefficients indicate a change in the retinal variables when the combined lifelong cumulative insulin exposure increases by 1 SD. The use of this lifelong cumulative insulin AUC addresses our hypothesis that both childhood and adulthood cumulative insulin are associated with retinal microvasculature, without the estimation of weights. Similarly, lifelong AUCs were constructed to other continuous cardiovascular risk factors. To assess lifelong smoking status, 3 groups were formed: nonsmokers, daily smokers either in childhood or adulthood, and daily smokers both in childhood and adulthood.

### Defining Participants With Clustering of Metabolic Risk Factors and Metabolically Healthy Controls

To study if, similarly to the effects of insulin on microvasculature in experimental settings (7, 8), the association of cumulative insulin differs by metabolic status, a similar approach as in Jahn et al was utilized (7); participants having clustering of metabolic risk factors (“metabolic syndrome”) in 2011 [definition according to the harmonized criteria of metabolic syndrome (30)] and who were nonsmokers and had no antihypertensive medication were identified. These participants formed the “metabolic syndrome” group.

To form a control group defined as metabolically healthy, participants who in 2011 had no metabolic syndrome, had BMI < 25 kg/m^2^, were nonsmokers, and had no antihypertensive or lipid-lowering medication and had no first-degree relatives with type 2 diabetes were identified. The use of antihypertensive and lipid-lowering medications and diabetes status of first-degree relatives were self-reported.

### Statistical Analysis

All variables were checked for normality, and variables with right-skew distribution were transformed using natural logarithmic transformation (arteriolar tortuosity, adulthood and childhood cumulative insulin, adulthood cumulative triglycerides and glucose).

Associations of standardized (mean 0, SD 1) childhood, adulthood, and lifelong cumulative insulin with the retinal microvascular measures were studied using linear models. Primarily, the models were fitted with sex and age as confounders. Second, the models were further adjusted for contemporary smoking status, systolic blood pressure, BMI, glucose, LDL-cholesterol, HDL-cholesterol, and triglycerides to control for conventional cardiovascular risk factors. Additionally, we tested the possible effect modification caused by sex on the associations between adulthood cumulative insulin and the retinal variables.

As an additional analysis, we tested the possible effect modification caused by metabolic status on the association between adulthood cumulative insulin and retinal variables.

To illustrate insulin levels across the lifespan by retinal arteriolar diameter in adulthood (aged 34-49 years), the participants were allocated into arteriolar diameter quartiles. In the illustration, the highest and lowest arteriolar diameter quartiles were applied.

The data analyses were performed with SAS 9.4 (SAS Institute, Inc., Cary, NC). Statistical significance is referred to as *P* < .05.

## Results

Characteristics of the study population are shown in Table 1. In 2011, 55.8% of the study participants were females and 18.9% were obese. Characteristics of cumulative adulthood and childhood insulin and other cardiovascular risk factors AUC are shown in Supplementary Table S1 (31). Two hundred forty-nine participants were smokers in childhood and 148 in adulthood. More detailed characteristics of adulthood and childhood insulin AUCs and participants included in this study vs those nondiabetics who participated in the 2011 follow-up but were excluded due to missing retinal or insulin data are shown in Supplementary Tables S2 and S3 (31). When compared to the excluded individuals, participants of the present study were older, were less frequently smokers, and had more often lipid-lowering medication. Additionally, participants of the 2011 follow-up with no retinal data had higher glucose levels when compared to the participants of the present study (Supplementary Table S3) (31).

**Table 1.** Characteristics of the study participants at the time of retinal assessment (follow-up year 2011)

	2011 (n = 1166)
Sex (% female)	55.8
Age (years)*^a^*	42 (34, 49)
Daily smoker, n (%) [total n]	148 (13.3) [1113]
Medication for hypertension, n (%) [total n]	98 (8.9) [1104]
Medication for cholesterol, n (%) [total n]	42 (3.6) [1166]
Systolic blood pressure (mmHg) [total n]	118.4 (14.1) [1162]
Diastolic blood pressure (mmHg) [total n]	75.0 (10.5) [1162]
BMI (kg/m^2^) [total n]	26.2 (4.7) [1161]
Obese, BMI > 30 kg/m^2^ (%)	18.9
Insulin (mU/L)*^b^* [total n]	7.3 (4.3, 10.7) [1166]
Total cholesterol (mmol/L) [total n]	5.2 (1.0) [1166]
HDL-cholesterol (mmol/L) [total n]	1.3 (0.3) [1165]
LDL-cholesterol (mmol/L) [total n]	3.3 (0.8) [1141]
Triglycerides (mmol/L)*^b^* [total n]	1.1 (0.8, 1.5) [1166]
Glucose (mmol/L) [total n]	5.3 (0.5) [1166]
Retinal measures	
Arteriolar diameter, pixels	17.9 (1.8)
Arteriolar tortuosity*^b^*	0.032 (0.012, 0.041)
Venular diameter, pixels	20.2 (2.6)
Venular tortuosity	0.013 (0.0086)

Values are means (SD) or counts (%)

All laboratory measures were analyzed from fasting serum samples.

Abbreviations: BMI, body mass index; HDL, high-density lipoprotein; LDL, low-density lipoprotein.

^*a*^Mean and range.

^*b*^Median and Q1 and Q3.

### Associations of Adulthood, Childhood and Combined Lifelong Cumulative Insulin With Retinal Measures

Cumulative adulthood, childhood (age 6-18 years) and lifelong insulin, respectively, were consistently and inversely associated with decreased arteriolar diameter when adjusted for age and sex. No other association between insulin AUCs and retinal measures were detected (Table 2). Therefore, we focused the following analyses on the retinal arteriolar diameter.

**Table 2.** Associations of standardized adulthood, childhood, and combined lifelong cumulative insulin with retinal measures

		Arteriolar diameter	Log arteriolar tortuosity	Venular diameter	Venular tortuosity
Model 1					
Cumulative adulthood insulin	Estimate (95% CI)	−0.18 (−0.28, −0.082)	0.035 (−0.014, 0.083)	−0.065 (−0.22, 0.085)	0.00031 (−0.00019, 0.00081)
	*P*-value	.0004	.16	.39	.23
Cumulative childhood insulin	Estimate (95% CI)	−0.31 (−0.50, −0.11)	0.047 (−0.047, 0.14)	−0.051 (−0.34, 0.24)	−0.00018 (−0.0011, 0.00077)
	*P*-value	.0019	.33	.73	.70
Cumulative combined life-long insulin	Estimate (95% CI)	−0.23 (−0.35, −0.12)	0.042 (−0.016, 0.099)	−0.070 (−0.25, 0.11)	0.00023 (−0.00036, 0.00081)
	*P*-value	.0001	.15	.44	.45
Model 2					
Cumulative adulthood insulin	Estimate (95% CI)	−0.23 (−0.38, −0.075)	*	*	*
	*P*-value	.0036	*	*	*
Cumulative childhood insulin	Estimate (95% CI)	−0.25 (−0.47, −0.035)	*	*	*
	*P*-value	.023	*	*	*
Cumulative combined life-long insulin	Estimate (95% CI)	−0.27 (−0.43, −0.11)	*	*	*
	*P*-value	.0012	*	*	*

Adulthood: Model 1 adjusted for age and sex. Model 2 further adjusted for adulthood smoking and cumulative adulthood BMI, systolic blood pressure, LDL-cholesterol, HDL-cholesterol, triglycerides, and glucose.

Childhood: Model 1 adjusted for age and sex. Model 2 further adjusted for childhood smoking and cumulative childhood BMI, systolic blood pressure, LDL-cholesterol, HDL-cholesterol, triglycerides, and glucose.

Combined life-long: Model 1 adjusted for age and sex. Model 2 further adjusted for combined lifelong smoking and cumulative combined lifelong BMI, systolic blood pressure, LDL-cholesterol, HDL-cholesterol, triglycerides, and glucose.

Abbreviations: BMI, body mass index; CI, confidence interval; HDL, high-density lipoprotein; LDL, low-density lipoprotein.

^*^Not included in the analysis due to the non-significant association in model 1.

When the analyses were further adjusted with contemporary smoking status, cumulative LDL-cholesterol, HDL-cholesterol, triglycerides, and glucose, all adulthood, childhood, and lifelong cumulative insulin persisted to associate with decreased arteriolar diameter (Table 2). Associations of the cross-sectional (year 2011), 4-year prior (2007), and 10-year (2001) prior adulthood insulin with retinal measures are shown in Supplementary Table S4 (31).

There was no effect modification caused by sex on the associations between adulthood cumulative insulin and the retinal variables, and therefore the analyses were not conducted separately by sex (data not shown).

### Effect Modification Caused by Metabolic Status on the Association Between Adulthood Cumulative Insulin and Arteriolar Diameter

Of the study participants, 144 were classified as having clustering of metabolic risk factors (“metabolic syndrome”) and 216 were classified as metabolically healthy. There was no significant effect modification caused by metabolic status on the association between cumulative adulthood insulin and arteriolar diameter (*P*-value for interaction .72), suggesting similar associations between the groups.

### Lifelong Insulin Classified by Retinal Arteriolar Diameter in Adulthood

**Figure 2.**
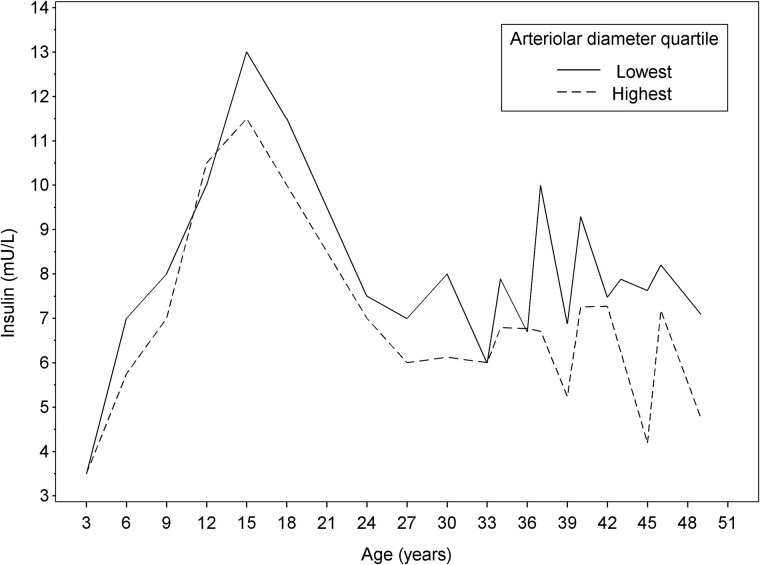
Median insulin levels across the lifespan among those study participants in the highest and lowest arteriolar diameter quartiles.

Figure 2 depicts insulin levels across lifespan among those in the highest vs lowest arteriolar diameter quartile. Participants in the lowest arteriolar diameter quartile appeared to have higher insulin levels across the lifespan, beginning from early childhood.

## Discussion

We found that exposure to higher levels of insulin during both childhood and adulthood was associated with narrower retinal arterioles in adulthood. Similarly, combined lifelong insulin exposure was associated with decreased arteriolar diameter. These associations remained after the adjustments for cumulative conventional cardiovascular risk factors. The results are in line with our recent observations in the STRIP cohort showing a trend toward decreased retinal arteriolar diameter in young adulthood in individuals exposed to higher lifelong insulin levels since infancy (14).

Retinal arteriolar narrowing is reported to predict cardiovascular morbidity and mortality in both general and diabetic populations (11, 12, 32). Similarly, both hyperinsulinemia and insulin resistance are reported to predict cardiovascular events in nondiabetic populations (33) and a genetically predicted high insulin level is causally associated with cardiovascular morbidity (34).

Endothelial dysfunction caused by high insulin levels is hypothesized to act as a link between metabolic disorders and cardiovascular disease development (4). Furthermore, it is hypothesized that endothelial dysfunction of the microvasculature may be one of the first steps of the cascade leading to cardiovascular disease progression (5, 6), and early microvascular changes seem to provide insight into vascular health before large artery structure is altered (14). In line with this, microvascular function seems to be altered early in the presence of insulin resistance (9, 35). Most of the studies on the effects of insulin on microvascular function are conducted on skeletal muscle microvasculature (9). Regarding the microvasculature of the eye, similar results are, however, reported: in a healthy population, the infusion of insulin increases blood flow in the ophthalmic artery and choroidal arteries (36-38). Furthermore, a trend toward increased retinal blood flow in response to insulin infusion has been shown (38), whereas in people with type 2 diabetes, insulin is reported to decrease retinal blood flow (39).

Regarding the microvasculature of the heart, insulin resistance is associated with conditions such as myocardial ischemia with no obstructive coronary arteries and heart failure with preserved ejection fraction—conditions that are characterized by coronary microvascular dysfunction (40, 41). Interestingly, retinal arteriolar narrowing is previously associated with decreased myocardial blood flow (42), and it predicts an increase in left ventricular size and mass and incident heart failure (43) even though some of these associations are lost when further adjusted for cardiovascular risk factors (43). These findings, however, are in line with the hypothesis that adverse microvascular changes of 1 organ is reflective of adverse systemic changes (5, 6), and insulin resistance seems to have a role in the pathogenesis of microvascular dysfunction (9).

Previous studies have indicated that in metabolically healthy individuals, insulin has a vasodilatory effect on microvasculature, whereas in the presence of insulin resistance and metabolic syndrome, insulin is reported to have a mainly vasoconstrictive effect on the microvasculature (4, 9). Insulin-mediated microvascular vasodilation seems to be due to nitric oxide (NO) synthesis, and vasoconstriction is related to insulin-stimulated secretion of endothelin-1 (ET-1) (4, 9). It is hypothesized that insulin resistance may cause an imbalance between NO and ET-1 release, leading to vascular vasoconstriction (4, 9), and both attenuated NO synthesis and increased ET-1 levels seem to be related already in the presence of early insulin resistance (35). In this study, a difference in the association between cumulative adulthood insulin and retinal arteriolar diameter by metabolic status was not detected, suggesting that, despite the metabolic status, higher cumulative insulin exposure is associated with decreased arteriolar diameter. In diet-induced insulin resistance, microvascular dysfunction is reported to manifest before systemic insulin resistance (35), and it can be hypothesized that this early microvascular insulin resistance and dysfunction might explain the findings of this study. However, in addition to functional changes, the microvasculature is prone to structural changes, such as arteriolar narrowing (41), and a fundus photo cannot distinguish between functional and structural microvascular changes. Indeed, the results of this study might also be explained by structural changes of the microvasculature associated with long-term insulin exposure and thus reflect a different phenomenon than previous experimental studies on exogenous insulin. In addition, insulin resistance-related pathogenic factors, such as inflammation, might have a role related to both increased insulin levels and decreased arteriolar diameter (4). Furthermore, the effect modification analysis included approximately 31% of all study participants, which might limit the statistical power.

In the present study, cumulative insulin was not associated with venular tortuosity, while our previous study showed an association between cumulative childhood and young adulthood (age 0-26 years) insulin with more tortuous venules (14). In addition, in a large-scale study, type 2 diabetes and metabolic risk factors such BMI and C-reactive protein are associated with increased venular tortuosity (17). When we consider that different methods were used to assess tortuosity between the present and our previous study, the difference between the results might be due to methodological issues. However, it might also be that early-life insulin exposure is associated with young adulthood venular tortuosity, whereas in mid-adulthood the association might be blunted by related cardiometabolic risk factors such as obesity and inflammation.

In addition to the strengths of this study, especially its longitudinal design extending from childhood to adulthood, we are also aware of its limitations. Fundus photos were captured only once, and therefore we were unable to assess longitudinal retinal changes. Fundus photo-derived microvascular measures cannot distinguish between structural and functional impairments of the microvasculature. Even though insulin was strongly associated with arteriolar diameter, this study might be unpowered to detect other possible associations. To utilize the maximum amount of available longitudinal data, different methods were used to construct adulthood and childhood AUCs. Due to the different applied AUC methodologies, the estimates are not directly comparable. Additionally, results of this study may be at least partly dependent on the method used to assess retinal measures. The population of the YFS comprises White participants, and the results of this study may not be applicable to other ethnicities.

## Conclusion

Cumulative adulthood, childhood. and combined lifelong insulin exposures are associated with decreased retinal arteriolar diameter in adulthood in a population without diabetes, even when adjusted for conventional cardiovascular risk factors.

## Data Availability

Due to the local legal restrictions concerning the distribution of all personal information, allowance of open access to the YFS data is not possible. Therefore, data sharing outside the study group requires a data-sharing agreement. Investigators can submit an expression of interest to the YFS Steering Group/Data Sharing Committee (PI of the YFS olli.raitakari@utu.fi).
